# Anti-biofilm and anti-glucosyltransferase effects of nano liposomal plant extracts against *Streptococcus mutans*

**DOI:** 10.1038/s41598-024-78728-1

**Published:** 2024-11-08

**Authors:** Faraz Radmand, Milad Baseri, Mohammad Yousef Memar, Alireza Ebrahimi, Hamed Hamishehkar, Solmaz Asnaashari, Amirreza Naseri, Maryam Kouhsoltani

**Affiliations:** 1 Student Research Committee, Faculty of Dentistry, Tabriz University of Medical Sciences, Tabriz, Iran; 2 Infectious and Tropical Diseases Research Center, Tabriz University of Medical Sciences, Tabriz, Iran; 3 Student Research Committee, Nutrition Research Center, Department of Food Science and Technology, Faculty of Nutrition and Food Sciences, Tabriz University of Medical Sciences, Tabriz, Iran; 4 Drug Applied Research Center, Tabriz University of Medical Sciences, Tabriz, Iran; 5 Biotechnology Research Center, Tabriz University of Medical Sciences, Tabriz, Iran; 6 Faculty of Dentistry, Zanjan University of Medical Sciences, Zanjan, Iran; 7 Department of Oral and Maxillofacial Pathology, Faculty of Dentistry, Tabriz University of Medical Sciences, Tabriz, Iran

**Keywords:** Nanoliposome, *Streptococcus mutans*, Plant extract, Biofilm, Glucosyltransferase, Microbiology, Nanoscience and technology

## Abstract

The role of *Streptococcus mutans* in the initiation of caries is related to its acidogenicity, aciduricity, and polysaccharides extracellular layer production by glucosyltransferases in dental biofilms. Therefore, inhibition of glucosyltransferase activity impairs the virulence of cariogenic biofilms, which can be used to prevent dental caries. We evaluated the anti-bacterial, anti-biofilm, and anti-glucosyltransferases effects of nanoliposomal herbal aqueous extracts of Liquorice (Glycyrrhiza glabra; G. glabra), Ginger (Zingiber officinale; Z. officinale), Pomegranate (Punica granatum; P. granatum), and Rose (Rosa damascene; R. damascene) via minimum bactericidal concentration and minimum inhibitory concentration against *Streptococcus mutans* strain ATCC 35,668. An anti-biofilm assay was performed using a minimum biofilm inhibitory concentration test. Among herbs, only P. granatum showed an antibacterial effect. Therefore, a nanoliposomal formulation of *P. granatum* was developed and characterized. Its effect on *S.mutans* glucosyltransferases was assessed by measuring glucan amount. The nanoliposomal formulation of P.granatum showed a significantly higher anti-biofilm effect than P. granatum aqueous extract. Their similar potential in blocking glucosyltransferases showed that the nanoliposomal formulation of P.granatum blocked other pathways rather than blocking glucosyltransferases for its anti-biofilm effect. Collectively, the nanoliposomal formulation of *P.granatum*, due to its anti- *Streptococcus mutans* characteristics, would be a production which open a new horizon for the oral pharmaceutical industry.

## Introduction

Dental caries is a common oral infectious disease globally which has a significant economic burden on the healthcare systems. Dental caries as an infectious disease has a considerable global economic impact e.g., in 2010 direct treatment corresponded to 4.6% of global health costs. In 2015, dental diseases cost the economy more than $544 billion USD. *Streptococcus mutans (S. mutans)* is the main cause of dental caries^1–3^. The *S. mutans* role in the initiation of caries is related to its acidogenicity and aciduricity^4^. *S. mutans* also produce an extracellular layer of polysaccharides in dental biofilms which are the glucosyltransferases (GTFs) products^5^. Extracellular polysaccharides, especially glucans formed by *S. mutans* GTFs, cause dental biofilm cariogenicity^6,7^. Thus, suppressing GTF activity as well as the subsequent synthesis of polysaccharides can impair the virulence of cariogenic biofilms, which can be used to inhibit the biofilm-induced disease^8,9^. There are many GTF inhibitors in natural products as the main and largely unknown source of GTF inhibitors, such as flavonoids, polymeric polyphenols, catechin-based polyphenols, proanthocyanidin oligomers, and some other compounds derived from plants^7^. Natural products are used to develop new medicines. Several studies have assessed the antimicrobial effects of extracts and essential oils. For instance, previous research showed that lemon myrtle could inhibit *S. mutans* GTF activity and biofilm formation^10^. Also in another in-vitro study, *Punica granatum* flower demonstrated an anti-GTF effect by analyzing the gene expression method^11^. Plant extracts have essential properties such as antioxidants and antimicrobials^12,13^. Such compounds exhibit potential therapeutic effects, but are restricted because of their poor solubility and stability. Drug delivery systems can be used as technological solutions to overcome these drawbacks. Nanoliposomes (NLPs) have been showing remarkable features as carriers of plant extracts because they are biodegradable and increase transcellular and paracellular drug transport^14,15^. Moreover, as lipid-based delivery systems, NPLs contain phospholipids, such as sphingomyelins and glycerophospholipids, allowing for the encapsulation of both lipophilic and hydrophilic constituents with their cell-resembling and self-assembling behavior^16,17^.

Although the wealth of literature published on the effect of various plant extracts on the control of dental caries, there is not enough focus on their anti-GTF effects. The aqueous plant extracts were investigated in this study which are appropriate for application in dental hygiene products and showed comparable anti *S.mutans* effects with alcoholic extracts^18^. Besides, aqueous plant extracts do not have dangerous side effects of alcoholic-based mouthwashes such as carcinogenicity^19,20^. Therefore, we evaluated the anti-bacterial, anti-biofilm, and anti-GTF effects of aqueous plant extracts of *Licorice* (*Glycyrrhiza glabra; G. glabra*), *Ginger (Zingiber officinale; Z. officinale)*,* Pomegranate (Punica granatum; P. granatum)*, and *Rose (Rosa damascene*; *R. damascene*) with a focus on the potential application of nanoliposomal delivery systems towards this issue. Our results may pave the way for the developing of new dental hygiene products and open a new horizon for further studies on GTF which seems to play an undeniable role in dental caries.

## Methods

### Materials

*S. mutans* strain ATCC 35,668 was obtained from the Iranian Research Organization for Science and Technology (IROST, Iran) and grown in tryptic soy broth (TSB) (Liofilchem, Italy). *G. glabra*,* Z. officinale*,* P. granatum*, and *R. damascene* were purchased from authentic medicinal plant stores. Mueller–Hinton Broth (MHB) (Liofilchem, Italy), Muller Hinton agar (MHA) (Biomaxima, Lublin, Poland), Chlorhexidine 0.2% (CHX) (Behsa, Tehran, Iran) and Sodium Fluoride (NaF) (Behsa, Tehran, Iran) were used for bacterial assays. Lecithin and cholesterol were received from VAV Lipids Pvt. Ltd. (Maharashtra, India) and Merck Chemicals Co. (Darmstadt, Germany). 

### Bacteria and cell growth

*S. mutans* strain ATCC 35,668 was obtained from the IROST and grown in TSB with 5% defibrinated sheep blood at 37 ◦C in 5% CO2 for 24 h. The organism was stored in TSB containing 20% glycerol at − 80 °C.

### Preparation of plant extract

Four herbal plants were purchased from an authentic medicinal plant store, including G. glabra, Z. officinale, P.granatum, and R.damascene. Ten grams of herbal plants were powdered, added to 100 mL of distilled water (DW), and kept at 37◦C for 24 h. After filtering through a bleached cotton cloth, the filtrate was lyophilized in a Christ Alpha1-4 freeze-dryer (Germany). The freeze-dried powder was dissolved in proper concentration in sterile distilled water and filtered through 0.45 μm pore-size membrane filter before assessing biological experiments.

### Antibacterial effect

The extract antibacterial effect was assessed by determining the minimum inhibitory concentration (MIC) by the broth microdilution technique in Cation-Adjusted Mueller–Hinton Broth (CAMHB)^21^. We considered MIC the lowest level of a compound that fully inhibits bacterial growth, as seen by the naked eye. Sterilized water was used as the negative control and CHX and NaF mouthwash as the positive controls of antimicrobial activity, respectively based on previous studies^22,23^. The minimum bactericidal concentration (MBC) was assessed by inoculating the wells used for MIC determination on the agar plates. MBC was regarded as the lowest compound concentration, killing 99.9% of the tested microorganisms.

### Encapsulation of extracts into NLPs

Among the herbs, only *P. granatum* showed an antibacterial effect. Therefore, the nanoliposomal formulation of *Punica granatum (P-*NLP*)* was prepared by the thin-film hydration method^24^, with some modifications. For this purpose, 90 mg lecithin and 10 mg cholesterol were dissolved in ethanol 96% (15 mL), and the dissolution process was continued on a stirrer for 20 min. Then, the mixture was poured into a round-bottom flask and underwent a thin film generation and solvent evaporation process by a rotary evaporator (Laborota 4002, Germany). Afterward, the lipid film was hydrated with a phosphate-buffered saline (PBS) of 2 mg/mL of the extract, stirring and vortexing for 2 min. The solution was processed by rotary evaporation at 60 °C (more than phase transition temperature) for 10 min, followed by a 2-min mixing process at room temperature. Then, to reduce the size of the particles formed, the mixture was sonicated by a UP200H ultrasound probe (Germany) with a nominal frequency of 50–60 Hz at 70% amplitude and 0.5 cycles for ten steps (1 min on and 0.5 min off). The generated NLPs were filtered through 0.45 μm pore-size membrane filter and kept in the refrigerator until assessment. After the characterization of P-NLP, the antibacterial tests were done with P-NLP and blank NLPs.

### Characterization of P-NLP

Dynamic Light Scattering (DLS) (Zetasizer Nano-ZS, UK) was used to measure P-NLP’s zeta potential and particle size at 25 °C in three replicates. Dilution of the P-NLP was done using double DW before the assessment. The morphology and shape of P-NLP were observed by scanning electron microscopy (SEM; EM3200, KYKY Instruments, China). The glass slide was coated with a specimen drop and left to air dry at room temperature. Then, using the direct current sputtering technique (DST1, Nanostructured Coating Co., Iran), it was coated with gold (100–150 Å) in an argon atmosphere before being observed. To determine the loading capacity (LC) and encapsulation efficiency (EE) of P-NLP, P-NLP (1 ml) was placed in the Amicon^®^ filter (molecular weight cut-off 100 kDa, UK), followed by dilution with DW. Then, the unloaded extract was separated from P-NLP by centrifugation at 3500 rpm for 10 min (Universal 320 centrifuge, Germany). Finally, the unloaded extract concentration loaded in P-NLP was assessed using UV-Vis spectroscopy (Ultrospec 2000, UK) at 255 nm, respectively. EE and LC were calculated using the following equations:

$$\begin{aligned}& \text{Encapsulation of efficacy}\:\left({\%}\right)\\ &\quad=\frac{\text{Total within nanoliposomal disperions}\:\left(\text{mg}\right)-\:\text{Free content}\:\left(\text{mg}\right)}{\text{Total extract within nanoliposomal disperions}\:\left(\text{mg}\right)}\times 100\\ &\text{Loading Capacity}\:\left({\%}\right)\\ &\quad=\frac{\text{Total within nanoliposomal disperions}\:\left(\text{mg}\right)-\text{Free content}\:\left(\text{mg}\right)}{\text{Total lipid content}\:\left(\text{mg}\right)}\times 100 \end{aligned}$$

### Biofilm inhibitory effect

The biofilm inhibitory effect of an aqueous extract of P. granatum and P-NLP was assessed by the minimum biofilm inhibitory concentration (MBIC). Firstly, 100 µL of 0.5 McFarland microbial suspensions were mixed with microplate wells containing 100 µL of TSB. Biofilm generation was done by adding 0.5% glucose to TSB. The suspension bacteria microplate was incubated at 35 °C for 24 h. After this time, the serial levels of tested compounds were transferred to wells, and microplates underwent incubation for 24 h at 37 °C. The contents were removed, and the wells were washed with sterile water and shaken vigorously. The optical density (OD) at 650 nm was measured on a microplate before and following incubation at 35 °C for 6 h. The MBIC was the lowest concentration of a compound that lid in an OD 650 variation at or below 10% of the mean of two positive control wells’ OD^25^.

### Anti-GTF effect

The GTFs were prepared to evaluate the effect of compounds on their activity as previously described^26^. To detect the GTF activity, alkali-soluble polysaccharide/glucan (ASP) generation and the water-soluble polysaccharide/glucan (WSP) level were determined by crude GTFs. Cell-free enzymes were precipitated from *S. mutans* culture supernatants by adding of solid ammonium sulfate to 70% saturation and recovery. It was kept at -70 °C and applied to generate water-insoluble and soluble glucan. The standard reaction mixture included crude enzyme (0.25 mL) and sub-MIC levels of antimicrobial compounds in phosphate buffer (20 mM; pH = 6.8), composed of 0.4 M sucrose (0.25 mL). The mixture underwent incubation at 37 °C for 18 h. The Phenol–sulphuric acid method measured the total amount of water-soluble and alkali-soluble polysaccharides (glucans). Three replicates were considered for each level of the compounds and their combination.

### Cell viability test

In all of the experimental steps, the cell viability test was done. For this purpose, bacteria cells were incubated with the sub-MIC concentration of tested compound dispersions in isotonic saline solutions at 37 °C for 1 h. The decrease in bacterial cell viability was determined using the colony counting technique. In brief, a series of 10-fold dilutions of bacterial suspension (100 µL each) were cultured on the MHA plates, and incubated overnight at 37 °C. Colonies were calculated and compared with the number of colonies on the control plates to determine variations in bacterial growth suppression. An isotonic saline solution free from the tested agent was considered a control.

### Statistical analysis

We used SPSS 19 to analyze the differences between the experimental and non-treated control groups using a one-way analysis of variance (ANOVA) and the Tukey-Kramer test. A P-value of < 0.05 was considered significant.

## Results

### Anti-bacterial and anti-biofilm effects of compounds

**Fig. 1:**
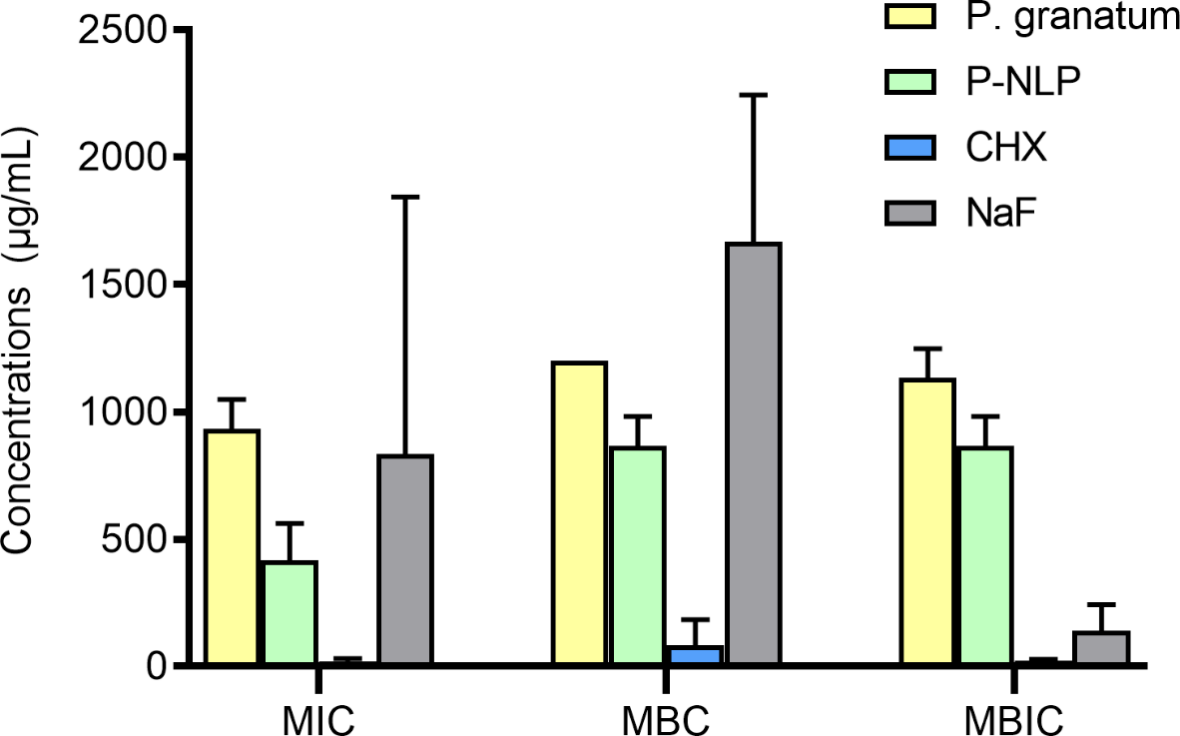
The MIC, MBC, and MBIC of tested compounds. Data are expressed as mean ± standard deviation (*n* = 3). MIC: minimum inhibitory concentration; MBC: minimum bactericidal concentration; MBIC: minimum biofilm inhibitory concentration; P. granatum: aqueous extract of *Punica granatum*; P-NLP: nanoliposomes of *Punica granatum* aqueous extract; CHX: chlorhexidine oral rinse; NaF: sodium fluoride oral rinse.

The aqueous extract of *G. glabra*,* Z. officinale*,* R. damascene*, and blank NLPs demonstrated no antibacterial activity and in three controlled repetitions, all concentrations (2–2048 µg/mL) showed bacterial growth. Therefore, they were abandoned from the continuing their work. The MIC, MBC, and MBIC results of the evaluated compounds are indicated in Fig. 1. According to our results, no significant difference was detected between groups in MIC (P-value = 0.667). In the MBC section, the results showed that *P*-NLP had approximately double potency against *S.mutans* compared to NaF. However, in the MBIC section, the NaF showed a significantly higher anti-biofilm effect than *P*-NLP (P-value < 0.05). Also, P-NLP had a higher anti-biofilm effect than the aqueous extract of *P. granatum*, which was statistically significant (P-value < 0.05).

### Characterization of NLPs

**Fig. 2:**
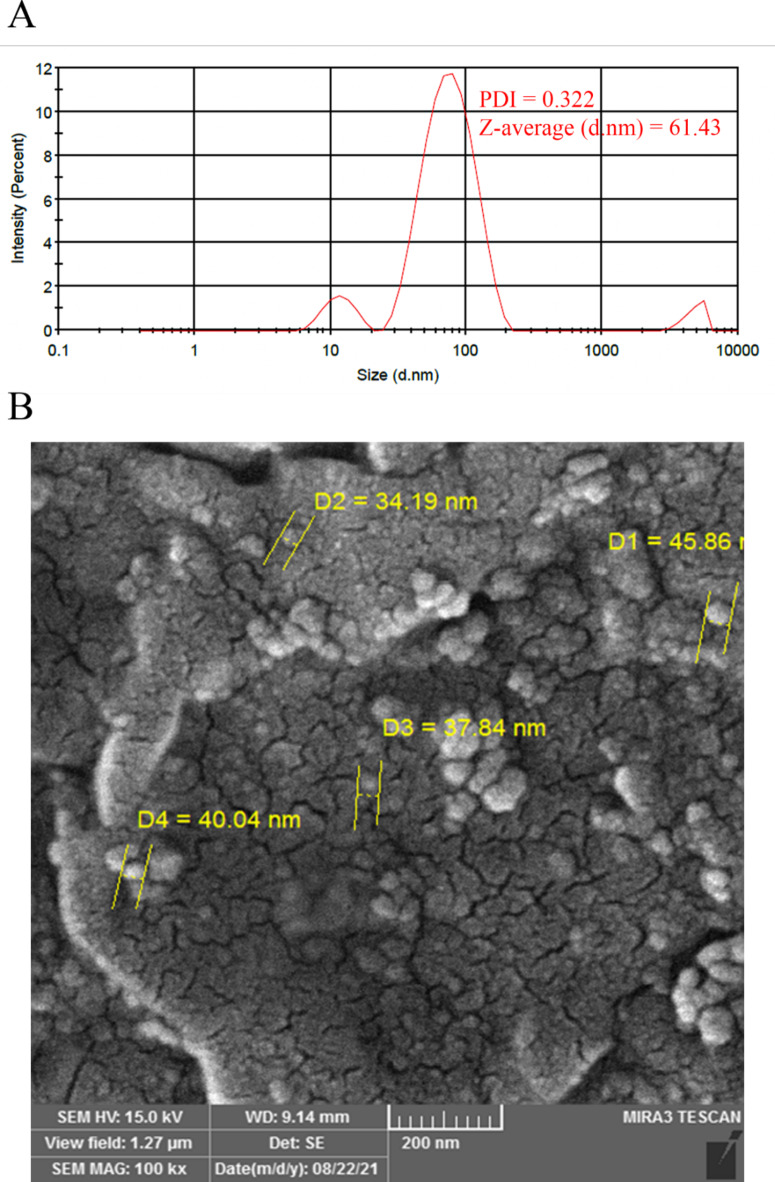
The particle size (**A**), and scanning electron microscopy image (**B**) of Nanoliposomes of *Punica granatum* aqueous extract.

As shown in Fig. 2a, the mean particle size and polydispersity index (PDI) of the P-NLP were 79.69 ± 32.40 nm and 0.322 ± 0.0672. The zeta potential was − 16.2 ± 6.68 mV and − 34.9 ± 7.81 mV for blank NPLs and P-NLP, respectively. The SEM image (Fig. 2b) of the P-NLP showed the narrow size distribution and spherical structure of liposomal particles. Also, most particles were smaller than 100 nm, which is consistent with the DLS results. The EE and LC values of the P-NLP were 92.29% and 18.79%, respectively.

### Anti-GTF effect

**Fig. 3:**
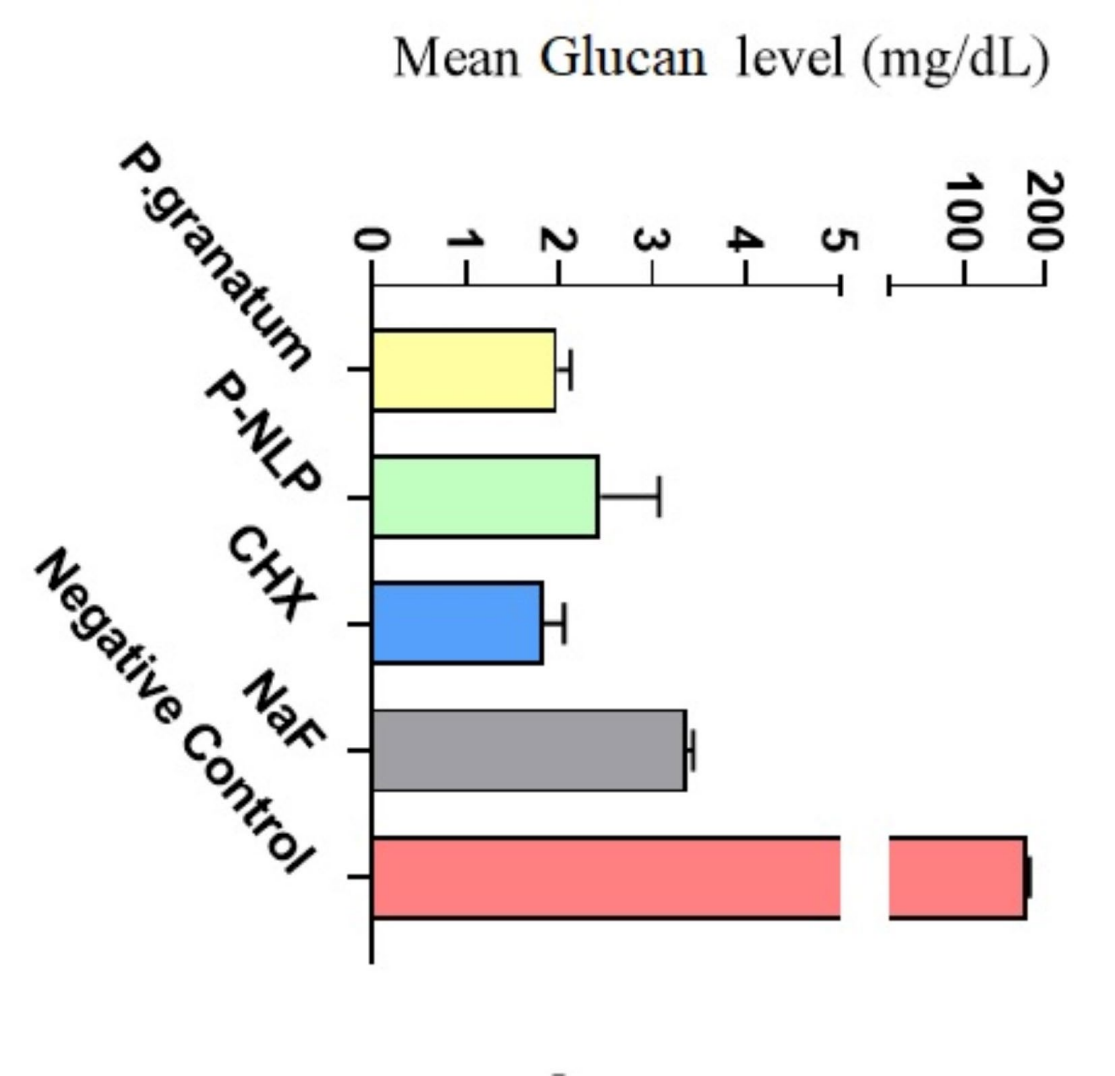
The Mean Glucan level of the evaluated compounds. Data is reported as mean ± standard deviation (*n* = 3). *P.granatum*: aqueous extract of *Punica granatum*; P-NLP: nanoliposomes of *Punica granatum* aqueous extract; CHX: chlorhexidine oral rinse; NaF: sodium fluoride oral rinse.

The results of the GTF enzyme activity assessment of tested compounds based on glucan levels are shown in Fig. 3. There was no statistically significant difference between groups in blocking GTF and glucan levels that mean Nano-liposomal formulation and aqueous extract of *P. granatum* and positive controls had significantly lower levels of glucan and GTF activity in comparison with the negative control group (*P-value* < 0.05).

### Cell viability test

In all of the experimental steps, the cell viability tests were done, and it showed that our compounds did not have bactericidal effects in the MIC and sub-MIC concentrations.

## Discussion

Considering the limitations of using alcoholic products for the daily and long-term use of dental hygiene, this study assessed the effects of aqueous herbal extracts on S. mutans. According to our hypothesis in this study, if GTF is blocked, only this important virulence factor of S. mutans will be inhibited without killing the bacteria. P. granatum and P-NLP inhibited the S. mutans and its GTF, and this inhibition was similar to conventional mouthwashes like CHX and NaF. MBC results showed that, unlike the CHX, our compounds did not disturb the bacterial ecosystem by preserving the S. mutans species.

The GTF enzyme is a key factor in biofilm formation that catalyzes the generation of insoluble- and soluble-linked glucans from sucrose and causes the dental plaque matrix polysaccharide composition^27^. According to our results, blocking the GTF solely would not lead to inhibiting biofilm formation, and this proves that for successful inhibition of biofilm formation, a holistic strategy is needed, which could halt the various virulence factors simultaneously.

Previous studies^28–31^ demonstrated that an aqueous extract of P. granatum peel inhibited the S. mutans successfully in different concentrations. The differences in the results of our study and the mentioned evidence were related to different methods of preparing extracts. Although a wealth of literature has been published on the effect of various herbal extracts on the control of dental caries, there is not enough focus on their anti-GTF effects, especially aqueous extracts, which are appropriate for application in dental hygiene products. Consistent with our result, Dastjerdi et al.^11^ showed that *P. granatum* aqueous extract at sub-lethal concentrations can down-regulate GTF genes. These results could be ascribed to P. granatum components like flavonoids, ellagitannins, and proanthocyanidin^32^.

Phospholipid-oriented delivery systems, such as liposomes are versatile assemblies that prevail against restrictions of plant extracts such as their poor solubility, stability, bioavailability, and gastric degradation^33^. NLPs are efficient carrier systems that deliver plant extracts with improved therapeutic impacts by increasing their limitations^34^. Our liposomes are a particular type called nano-phytosomes, which were used for plant extracts^35^. Their advantage is that not only were the extracts concentrated in the nanoliposomes, but they were also attached to the outer surface of the nanoliposomes. This could be explained by the uniformity of these particles according to the PDI result (< 0.5 μm)^36^, that they form a complex structure with bonds between a negative potential of extracts and the positive potential of colin of phospholipids that leads to the more negative potential of our liposomes. Therefore, the loading capacity of our nanoliposomes increased to 18.79%. However, our results showed that encapsulation of the P. granatum extract did not affect its anti-bacterial potential, considering the MIC, MBC, and GTF activity inhibition.

Although P. granatum and P-NLP showed significantly lower effectiveness against S. mutans biofilms than CHX, the P-NLP had significantly higher anti-biofilm effects than P. granatum. Zeta potential indicates a colloidal system’s physical stability by assessing the surface charge of particles^37^. Zeta values ± 30 mV can protect nanoparticles against reduction and aggregation of electrostatic repulsion between nano-phytosomes resulting in the generation of large aggregates of nano-phytosomes vesicles^38^. Therefore, one of the reasons that can explain this difference is that our NLP zeta potential which is below − 30 mV, helped to reach to higher stability of medicine^39^. The other reason would be the surface charge of the *S.mutans* and NLPs, which both of them are negative^40^, which may disturb the accumulation of these bacteria, obstructing biofilm formation.

In this study the effect of the aqueous extract of *R. damascene* on *S. mutans* was not effective. Previous studies about this plant extract showed significant antibacterial results on *S. mutans*^41–43^. This difference between studies may be due to the type of extract which was alcoholic in other studies rather than our aqueous extract, which can be due to more effective dissolving capacity in alcohol, higher bioavailability, or the polar nature of the alcoholic solvent, leading to the leaching of more active ingredients during extraction^44,45^.

*G. glabra* was the other herbal extract that was investigated in our study. Studies examining the effect of *G. glabra* extract on *S. mutans* revealed that alcoholic extracts are significantly more effective than aqueous ones. In this study, we did not find any remarkable effect of the aqueous extract of *G. glabra* on *S. mutans*, unlike previous studies^45,46^. This discrepancy may be due to different concentrations in MIC tests. Our maximum concentration was 2000 µg/ml but the MIC concentration in previous studies was much more than this.

*Z. officinale* aqueous extract did not show a significant effect on *S. mutans*. Evidence showed that both aqueous^47^ and alcoholic^48^ extracts had significant antibacterial activity against *S. mutans*^49,50^. Another study that evaluated the aqueous and alcoholic extracts of *Z. officinale* showed that both types of extracts were effective but the alcoholic one was more effective^51^. It is noteworthy that our method of assessing the antibacterial effect differed from the above studies, which may lead to controversial results.

The most noticeable strength of this study was using NLPs to carry the extracts. The study suggests that various extracts, including aqueous extract, could be employed in future studies to develop these compounds into mouthwash. Conducting in vivo evaluations of these medicines and assessing their impact on dental caries would provide robust and dependable evidence for patients’ continued use of these medicines. Also, some modifications in the production of NLPs and increasing their beneficial characteristics, such as stability and bioavailability, would increase their antimicrobial potential.

## Conclusion

P-NLP showed a significantly higher anti-biofilm effect compared to the aqueous extract of P. granatum. Their similar potential in blocking GTF showed that P-NLP has blocked pathways other than blocking GTF for its anti-biofilm effect. A promising anti-biofilm role was revealed in our study by applying P-NLP as a future anti-S. mutans ingredient of mouthwashes.

## Data Availability

The datasets used and analyzed in the current study available from the corresponding author on reasonable request.
